# The Role of Childhood Obesity in Acute Presentations and Outcomes of Hospitalized COVID-19 Patients

**DOI:** 10.7759/cureus.28911

**Published:** 2022-09-07

**Authors:** Tyler Tolopka, Joshua Kuehne, Kiran Mainali, Morgan Beebe, Melinda Garcia, Mohammed Salameh, Rosario Ocampo, Utpal Bhalala

**Affiliations:** 1 Pediatrics, University of the Incarnate Word School of Osteopathic Medicine, San Antonio, USA; 2 Epidemiology and Public Health, University of the Incarnate Word School of Osteopathic Medicine, San Antonio, USA; 3 Pediatrics, Baylor College of Medicine, Houston, USA; 4 Pediatric Medicine, University of Texas Health Science Center at San Antonio, San Antonio, USA; 5 Anesthesiology and Critical Care, University of Texas Medical Branch at Galveston, Galveston, USA; 6 Anesthesiology and Critical Care, Driscoll Children's Hospital, Corpus Christi, USA

**Keywords:** outcomes, body mass index, obesity, sars-cov-2, covid-19, children

## Abstract

Background and objective

The severe acute respiratory syndrome coronavirus 2 (SARS-CoV-2), which causes coronavirus disease 2019 (COVID-19), has affected all regions, demographics, and age groups worldwide. However, few studies have investigated the prevalence of childhood obesity and severe COVID-19 presentation in a predominately Hispanic population. In light of this, we investigated the role of underlying obesity in COVID-19 presentations and outcomes at a tertiary care children’s hospital by using subcategories based on patients' body mass index (BMI).

Methods

We conducted a single-center retrospective study involving 77 pediatric patients aged 18 years and younger, who were hospitalized with suspected or verified COVID-19 between February 2020 and January 2021. We collected data on height, weight, and BMI and categorized patients based on the World Health Organization (WHO) and Centers for Disease Control and Prevention (CDC) definition(s) of obesity. We also collected data on demographics, mode of presentation, need for pediatric intensive care unit (PICU) admission, the severity of illness at the time of PICU admission, and data related to outcomes. We analyzed the data using logistical regression with Firth’s biased reduction method wherever applicable.

Results

In our cohort, over 85% of the patients identified as belonging to Hispanic ethnicity (n=66); the median age of the cohort was 8.69 years, and 50.65% were classified as obese (n=39). We found a statistically significant relationship between underlying obesity and one or more comorbidities (p<0.001). BMI classification was significantly dependent on the incidence of multisystem inflammatory syndrome in children (MIS-C) (p=0.0353). Furthermore, the bivariate analysis confirmed that acute kidney injury (AKI) (p=0.048) and MIS-C predictors (p<0.001) were significantly associated with PICU admission status. The combined model confirmed a significant relationship between MIS-C and both PICU admission status (p<0.001) and obese BMI classification (p=0.002). PICU admission status led to increased hospital length of stay (LOS) (p<0.001). Patient age (p=0.003), underweight BMI (p=0.034), and obese BMI (P=0.008) were significant predictors of PICU LOS. Of note, the survival rate among admitted COVID-19 patients was 93.5%.

Conclusion

Based on our findings on the prevalence of underlying obesity in admitted COVID-19 patients at the Children’s Hospital of San Antonio, over 50% of pediatric patients were obese and predominately Hispanic. Obesity was significantly associated with patient age, comorbidities, MIS-C status, and PICU LOS. Hospital mortality in pediatric COVID-19 patients was low (6.49%) and consistent with other studies in the literature showing lower rates of mortality in children versus mortality in adult patients with COVID-19.

## Introduction

The ongoing coronavirus disease 2019 (COVID-19) pandemic, caused by the severe acute respiratory syndrome coronavirus 2 (SARS-CoV-2), has cost countless lives globally, affecting all regions, demographics, and age groups. Over the past year, clinical research has been paramount in identifying significant risk factors, important clinical presentation features, and potential therapeutic modalities to combat severe COVID-19 infection.

In adults, obesity is associated with immune dysfunction with increased SARS-CoV-2 viral load/shedding, leading to symptom severity [1]. As SARS-CoV-2 penetrates angiotensin-converting enzyme 2 (ACE2) receptors more copiously in adipose tissue (compared to lungs or respiratory tract), obese patients are predisposed to more severe illness [2]. Along with acute respiratory distress and hypoxia, obese COVID-19 adults are predisposed to systemic inflammation and a higher risk of thrombotic events, including venous thromboembolism [2]. These comorbid factors have led to worsening outcomes in COVID-19 illness including respiratory failure, the need for mechanical ventilation, and higher mortality [2]. In March of 2021, the Centers for Disease Control and Prevention (CDC) released a report comparing body mass index (BMI) classification in adults with characteristics of severe COVID-19 hospital presentation. BMI classifications of overweight and obese in adults were significant risk factors for the need for invasive mechanical ventilation, and obese adults with COVID-19 infection had higher rates of hospitalization, ICU admission, and mortality [3].

In addition to risk factors and comorbidities, ethnic and other minority statuses and low socioeconomic status have played a role in the impact of the COVID-19 pandemic [4]. Hispanics, the largest minority group in the United States, have accounted for 46.4% of the positive cases in Texas as of May 14, 2020 [4]. Hispanic ethnicity has been associated with higher rates of chronic comorbid disease including obesity, more severe COVID-19 infection, and a two-fold higher age-adjusted death rate compared to Whites [4]. Multiple chronic conditions, barriers to accessing healthcare, immigration status and exclusion from Medicaid coverage, language barriers, and financial burden are all significant issues faced by minority populations in the United States [4].

Currently, most data related to COVID-19 infection in children report mild symptoms for the most part [5-7], as derived from case reports, case series, small retrospective reviews, systematic reviews, and meta-analyses [1-11]. Obesity has been recognized as an independent risk factor for critical COVID-19 infection in children [12-14].

Retrospective studies that specifically focus on pediatric obesity and severe COVID-19 infection and outcomes among Hispanics are sparse. Therefore, this study looks to build upon these studies by analyzing presenting factors, illness severity markers, and outcomes in a predominately Hispanic population with a higher prevalence of obesity at a tertiary hospital in Southwest Texas. Due to the high, disproportionate prevalence of obesity among Hispanic children in San Antonio compared to national estimates, our study provides a unique perspective on the health disparities experienced by pediatric patients with underlying obesity within the context of the ongoing COVID-19 pandemic, with a view to improving (1) risk management strategies within intensive care and (2) prognostic value for acute illness presentation of the minority pediatric patients [15].

This article was previously presented as a meeting abstract at the 2022 National PAS Annual Scientific Meeting on April 24, 2022.

## Materials and methods

Study design and setting

We conducted a single-center retrospective study involving children aged 18 years and younger who were hospitalized with COVID-19 infection at the Children's Hospital of San Antonio, a tertiary care children’s hospital, between February 2020 and January 2021. We obtained institutional review board approval/exempt status, with a waiver for informed consent. Patients aged >18 years of age or surgical patients with incidental COVID-19 diagnosis were excluded. Data on height, weight, and BMI were collected from the electronic medical records (EMR); the World Health Organization (WHO) and CDC criteria and age-based growth charts were used to classify obesity [16,17]. BMI classification was then used to analyze its role in demographics, comorbidities, presenting factors, illness severity, and hospital outcomes. This methodology has been accepted and used in prior retrospective studies comparing pediatric BMI and pediatric intensive care unit (PICU) outcomes in the context of severe infection [18].

Illness severity

For patients admitted to the PICU, Pediatric Risk of Mortality III (PRISM III) and Pediatric Logistic Organ Dysfunction-2 (PELOD-2) scores were calculated to quantify illness severity. We classified illness severity into mild (PRISM III score <10 or PELOD-2 score ≤5), moderate (PRISM III score 10-19 or PELOD-2 score 6-12), and severe illness (PRISM III score >19 or PELOD-2 score >12) based on Goncalves et al. [19].

Outcome measure

Our primary outcome measure was PICU admission based on BMI classification. Secondary outcomes included the incidence of acute kidney injury (AKI) utilizing the Pediatric Risk, Injury, Failure, Loss, End Stage Renal Disease (pRIFLE) criteria, the incidence of multisystem inflammatory syndrome in children (MIS-C), the need for invasive mechanical ventilation, PICU length of stay (LOS), hospital LOS, and hospital mortality [20,21].

Statistical analysis

Descriptive statistics were used to summarize demographics and clinical presenting factors. We used the Kruskal-Wallis test to analyze the relationships between continuous dependent and categorical independent variables, while Fisher’s exact test was utilized to analyze the relationship between categorical variables [22,23]. The logistic regression model was employed to investigate the bivariate relationships between binary dependent and continuous/categorical independent variables. The significance of age, BMI classification, and other hospital outcomes was also considered based on potential predictive factors of severe illness. Furthermore, our combined model utilized the logistic regression model for multivariate analysis to check the relationships between binary dependent and multiple continuous/categorical independent variables based on these predictors. Our combined model selected the best model using the backward elimination process where variables with p<0.05 were retained. For the bivariate and multivariate analyses, the odds ratios (OR) and their 95% confidence intervals (CI) were calculated. Since the distribution of binary outcomes was unbalanced in our data, we utilized the logistic regression model with Firth’s biased reduction approach for both bivariate and combined models. Imputation and statistical analysis were computed using the R software (The R Foundation for Statistical Computing, Institute for Statistics and Mathematics, Vienna, Austria) [24].

## Results

**Figure 1:**
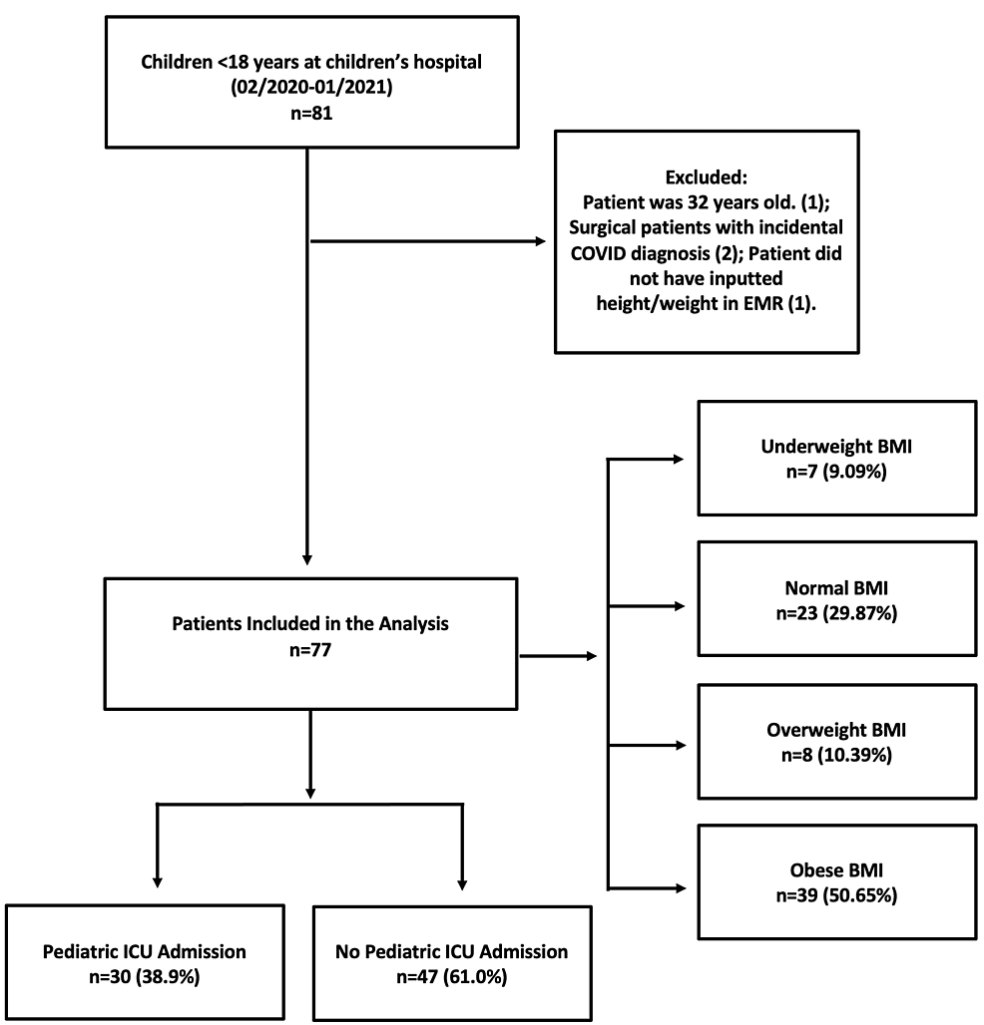
Consort diagram Consort diagram demonstrating the sample size of pediatric COVID-19 patients in the study BMI: body mass index; COVID-19: coronavirus disease 2019; EMR: electronic medical record

Our sample consisted of 77 pediatric patients (38 males and 39 females) admitted with COVID-19 infection at a tertiary care center (Figure 1).

Demographics, comorbidities, and presenting factors

**Figure 2:**
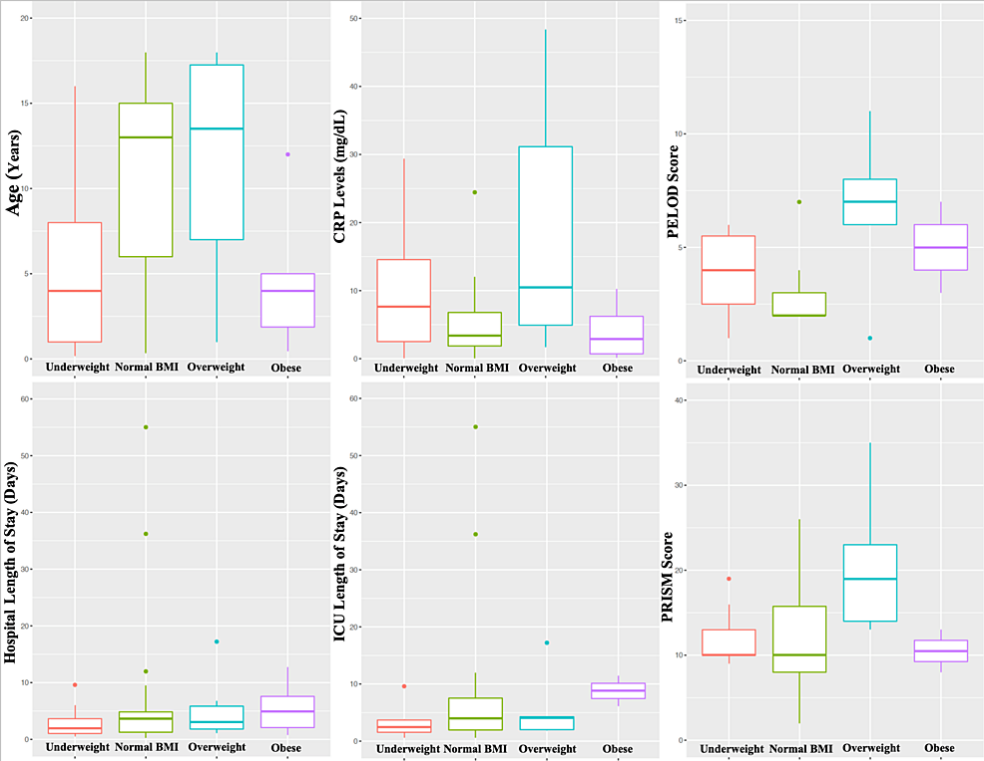
Outcomes Age distribution, CRP levels, hospital LOS, PICU LOS, and PELOD-2 and PRISM III scores by BMI classification in pediatric COVID-19 patients BMI: body mass index; COVID-19: coronavirus disease 2019; CRP: C-reactive protein; LOS: length of stay; PELOD-2: Pediatric Logistic Organ Dysfunction-2; PICU: pediatric intensive care unit; PRISM III: Pediatric Risk of Mortality III

Of note, 50.65% of pediatric patients in our cohort fulfilled the obese BMI classification criteria (n=39), while our normal BMI subsample included 23 patients for reference comparison. Significantly, over 85% of our patient sample identified as belonging to Hispanic ethnicity (n=66), and the prevalence of obesity was 54.5%. All demographic and predisposing factors are detailed in Table 1. There was a significant difference in age distribution among the four BMI categories (p=0.002). Specifically, differences were observed in age distributions of normal BMI compared to overweight patients (p=0.047) and normal BMI compared to obese patients (p=0.016), as displayed in Figure 2. The prevalence of one or more comorbidities within our sample had a significant dependent relationship with BMI status (p=1.34E-15). Further analysis showed a statistically significant difference in association with comorbidities of the following BMI categories: normal, obese BMI (p=1.04E-11); obese, overweight BMI (p=9.54E-09); obese, underweight BMI (p=3.74E-08). Even though Hispanic patients accounted for 85% of our sample, there was no significant relationship between race or ethnicity and BMI classification (Table 1).

**Table 1:** Demographic information Demographics and comorbidities by BMI classification in pediatric COVID-19 patients BMI: body mass index; COVID-19: coronavirus disease 2019

Demographics and predisposing factors						
								Total (n=77)	Underweight (n=7)	Normal BMI (n=23)	Overweight (n=8)	Obese (n=39)	P-value
Mean age (years)	8.69	4.31	5.38	10.52	10.68	0.002
Sex						0.4214
Male	49.4% (n=38)	5.3% (n=2)	36.84% (n=14)	7.9% (n=3)	50% (n=19)	
Female	50.65% (n=39)	12.8% (n=5)	23.1% (n=9)	12.82% (n=5)	51.28% (n=20)	
Race						0.642
White/Caucasian	68.8% (n=53)	7.55% (n=4)	30.19% (n=16)	7.55% (n=4)	54.72% (n=29)	
Black/African American	3.90% (n=3)		67% (n=2)		33.3% (n=1)	
American Indian/Alaska Native	2.6% (n=2)			50% (n=1)	50% (n=1)	
Other	24.68% (n=19)	10.53% (n=2)	31.58% (n=6)	15.79% (n=3)	42.11% (n=8)	
Ethnicity						0.1468
Hispanic	85.71% (n=66)	9.09% (n=6)	24.24% (n=16)	12.12% (n=8)	54.55% (n=36)	
Non-Hispanic	14.29% (n=11)	9.09% (n=1)	63.64% (n=7)		27.27% (n=3)	
Comorbidities						<0.0001
One or more	55.84% (n=43)		9.30% (n=4)		90.69% (n=39)	

Risk factors for critical illness

There was a high incidence of AKI, MIS-C, and patients requiring PICU admission in our sample (Table 2).

**Table 2:** Presentation Presenting factors and outcomes by BMI classification in pediatric COVID-19 patients AKI: acute kidney injury; BMI: body mass index; COVID-19: coronavirus disease 2019; MIS-C: multisystem inflammatory syndrome in children; PICU: pediatric intensive care unit

Presenting factors and outcomes						
								Total (n=77)	Underweight BMI (n=7)	Normal BMI (n=23)	Overweight BMI (n=8)	Obese BMI (n=39)	P-value
Binomial factors						
AKI	27.27% (n=21)	14.26% (n=1)	17.39% (n=4)	50% (n=4)	30.77% (n=12)	0.1955
MIS-C	14.29% (n=11)	14.29% (n=1)	21.74% (n=5)	37.5% (n=3)	5.13% (n=2)	0.0353
PICU admission	38.96% (n=30)	28.57% (n=2)	26.09% (n=6)	62.50% (n=5)	43.59% (n=17)	0.134
Mortality	6.49% (n=5)			25% (n=2)	7.69% (n=3)	0.0989

Of the 21 admitted patients who presented with COVID-19 and AKI, 57.14% were classified as obese, and 76.19% were classified as either overweight or obese. Using the pRIFLE criteria, the 21 patients with AKI were further classified as risk (n=6), injury (n=6), and failure (n=9). Of the patients who were classified as having renal failure, all were either overweight or obese. As shown in Table 3, AKI was a significant independent predictor of PICU admission status (p=0.048).

**Table 3:** Severity of illness Risk factors for critical illness in pediatric COVID-19 patients AKI: acute kidney injury; BMI: body mass index; CI: confidence interval; COVID-19: coronavirus disease 2019; LOS: length of stay; MIS-C: multisystem inflammatory syndrome in children; OR: odds ratio; PICU: pediatric intensive care unit

Severity of illness factors					
							Bivariate analysis (n=77)		Combined model (n=77)		
Predictors	OR (95% CI)	P-value	OR (95% CI)		P-value
AKI					
Age	1.009 (0.931-1.093)	0.829			
Underweight BMI	1.00 (0.114-8.737)	1			
Overweight BMI	4.33 (0.763-24.614)	0.081			
Obese BMI	1.97 (0.566-6.854)	0.264			
PICU admission status	2.73 (0.984-7.618)	0.048	2.73 (0.984-7.618)		0.048
MIS-C					
Age	0.997 (0.902-1.102)	0.953			
Underweight BMI	0.776 (0.092-6.548)	0.802	0.299 (0.012-7.26)		0.425
Overweight BMI	2.14 (0.382-11.98)	0.368	0.402 (0.032-5.041)		0.457
Obese BMI	0.224 (0.044-1.134)	0.054	0.046 (0.005-0.452)		0.002
AKI	1.69 (0.456-6.310)	0.8			
PICU admission status	56.02 (3.047-1030.15)	<0.0001	126.505 (5.932-2697.925)		<0.0001
Hospital LOS	1.021 (0.955-1.092)	0.484			
Mechanical vent status (reference is 0 or "No Vent")	0.486 (0.019-12.341)	0.2			
PICU admission status					
Age	1.049 (0.974-1.131)	0.194			
Underweight BMI	1.224 (0.194-7.736)	0.821			
Overweight BMI	4.231 (0.778-22.997)	0.0737			
Obese BMI	2.094 (0.686-6.394)	0.177			
AKI	2.738 (0.979-7.661)	0.048			
Hospital LOS	1.436 (1.159-1.780)	<0.0001	1.436 (1.159-1.780)		<0.0001
Mechanical vent status	2.316 (0.368-14.595)	0.325			

MIS-C affected 11 patients in our sample. BMI classification and MIS-C status were statistically dependent (p=0.0353), as detailed in Table 3. Furthermore, the bivariate analysis confirmed that MIS-C predictors were significantly associated with PICU admission status (p=5E-06). The combined model confirmed a significant relationship between MIS-C and both PICU admission status (p=4.42E-07) and obese BMI classification (p=0.002). Table 4 provides an analysis of Hispanic ethnicity on risk factors for critical illness, which revealed no significant findings.

**Table 4:** Ethnicity Hispanic ethnicity and risk factors for critical illness in pediatric COVID-19 patients AKI: acute kidney injury; CI: confidence interval; COVID-19: coronavirus disease 2019; MIS-C: multisystem inflammatory syndrome in children; OR: odds ratio; PICU: pediatric intensive care unit

Hispanic ethnicity and risk factors for critical illness		
			Predictors	OR (95% CI)	P-value
Hispanic ethnicity		
AKI (reference is 0 or "No AKI")	0.554 (0.053-3.048)	0.7146
MIS-C (reference is 0 or "No MIS-C")	2.67 (0.379-14.620)	0.1872
PICU admission status (reference is 0 or "No PICU")	0.881 (0.171-3.882)	1

Hospital course

Determining factors of hospital course are detailed in Table 5.

**Table 5:** Risk factors Determining factors of hospital course in pediatric COVID-19 patients *Measured in about 50% of admitted COVID-19 patients. **Includes two patients not admitted to PICU with invasive mechanical ventilation BMI: body mass index; COVID-19: coronavirus disease 2019; CRP: C-reactive protein; LOS: length of stay; PICU: pediatric intensive care unit

Determining factors of hospital course								
										Total (n=77)	Underweight BMI (n=7)	Normal BMI (n=23)		Overweight BMI (n=8)		Obese BMI (n=39)	P-value
Continuous factors								
CRP levels (mg/dL)*	8.56 (n=39)	4.05 (n=4)	10.27 (n=12)		19.32 (n=5)		5.42 (n=18)	0.2435
Mechanical ventilation**	6.49% (n=5)		16.67% (n=1)				5.88% (n=1)	0.1727
Hospital LOS (days)	4.76 (n=77)	5.4 (n=7)	2.7 (n=23)		5.02 (n=8)		5.8 (n=39)	0.282

In a subset of patients (n=39), CRP levels were measured at day 0 of hospitalization. No significant differences in CRP levels were observed among the four BMI categories, with their respective distributions shown in Figure 2.

Of the 39 acutely presenting COVID-19 pediatric patients in our sample with underlying obesity, 43.59% required PICU admission (n=17). When comparing all PICU-admitted patients (n=30), there was no significance between PICU admission status and variables including BMI classification, and PELOD-2 and PRISM III scores (Table 6). In our cohort, none of the patients needed dialysis or continuous renal replacement therapy (CRRT), and their renal function recovered completely with supportive care during the hospital course.

**Table 6:** Hospital course Hospital course for critically ill COVID-19 patients admitted to the PICU. We classified illness severity into mild (PRISM III score <10 or PELOD-2 score ≤5), moderate (PRISM III score 10-19 or PELOD-2 score 6-12), and severe illness (PRISM III score >19 or PELOD-2 score >12) *PRISM III scores given to n=37 patients: n=30 on the PICU floor and n=7 on the PIMC floor BMI: body mass index; COVID-19: coronavirus disease 2019; LOS: length of stay; PELOD-2: Pediatric Logistic Organ Dysfunction-2; PICU: pediatric intensive care unit; PRISM III: Pediatric Risk of Mortality III

Hospital course								
										Total (n=30)	Underweight BMI (n=2)	Normal BMI (n=6)		Overweight BMI (n=5)		Obese BMI (n=17)	P-value
Continuous factors								
PICU LOS (days)	7.39 (n=30)	8.79 (n=2)	3.76 (n=6)		5.86 (n=5)		8.95 (n=17)	0.2753
Mean PELOD-2 score	3.7 (n=30)	5 (n=2)	4.17 (n=6)		6.6 (n=5)		2.59 (n=17)	0.0842
Mean PRISM III score*	12.7 (n=37)	10.5 (n=2)	12.3 (n=8)		20.8 (n=5)		10.71 (n=24)	0.1128

Figure 2 shows the distribution of PELOD-2 and PRISM-III scores of PICU-admitted patients by BMI classification.

Outcomes

Though higher mean hospital LOS was seen in both overweight and obese BMI categories, there were no statistically significant differences in hospital LOS by BMI classification. However, PICU admission status was significantly associated with overall hospital LOS (p=1.5E-05), as shown in Table 3. For patients admitted to the PICU (n=30), age (p=0.003), obese BMI classification (p=0.008), and underweight BMI classification (p=0.034) were found to have a significant relationship with PICU LOS, as detailed in Table 7.

**Table 7:** Outcomes Outcomes in critically ill COVID-19 patients admitted to the PICU BMI: body mass index; CI: confidence interval; COVID-19: coronavirus disease 2019; LOS: length of stay; PICU: pediatric intensive care unit

Outcomes		
					Bivariate analysis (n=30)
Predictors	Coefficient estimate (95% CI)	P-value
PICU LOS		
Age	-1.385 (-2.256, -0.514)	0.003
Obese BMI (reference=normal BMI)	17.055 (4.726, 29.385)	0.008
Underweight BMI	16.901 (1.310, 32.492)	0.034
Overweight BMI	6.230 (-10.6726, 23.133)	0.458

Obese BMI was not found to have significance in terms of overall case mortality, and the survival rate from COVID-19 within our patient population was 93.5%.

## Discussion

Our findings contribute to the data on the impact of obesity and acute COVID-19 infection in pediatric patients. In the context of acute COVID-19 presentation, our pediatric sample contained an 85% Hispanic population, a high prevalence of obesity, and a significant difference in age distribution based on BMI classification. Obese patients in our sample were more likely to be older, have one or more comorbidities, present with MIS-C, and require PICU admission. These findings complement the results of recent meta-analyses on the prevalence of obesity in hospitalized COVID-19 pediatric patients [12,13], and its impact on critical illness and hospital course [13]. Although almost 40% of pediatric patients with acute COVID-19 presentation were admitted to the PICU, we were not able to show obesity as an independent risk factor for PICU admission status, invasive mechanical ventilation status, and quantitative PELOD-2 and PRISM III assessments. Underweight and obese BMI levels also led to a significantly longer LOS in the ICU, consistent with prior studies showing a J-curve association of pediatric BMI abnormalities with increased hospital and ICU LOS [13]. We also observed the independent significance of AKI and MIS-C on PICU admission, and these should continue to be appreciated in children with severe COVID-19 infection to improve hospital outcomes [8-12]. Overall mortality at our tertiary center was low at 6.49% (n=5), and hence the findings in our small sample were not able to better correlate the role of pediatric obesity in COVID-19 case mortality.

Bhalala et al. have published a large retrospective study that outlined the characteristics and outcomes of COVID-19 in pediatric patients [12]. In a cohort of 874 children, 62.9% identified as non-Hispanic, 46.2% required PICU admission, and there was a 36.35% prevalence of obesity among admitted children [12]. Another study (n=795) by Tripathi et al. showed that admitted COVID-19 patients had a 31.5% prevalence of obesity and they were also more likely to be diagnosed with MIS-C, have higher ICU admission rates, and increased hospital LOS [13]. They also recognized obesity as an independent risk factor for critical COVID-19 infection, with a more significant impact on pediatric outcomes than those diagnosed with MIS-C [13]. Finally, The Journal of Pediatrics published a related study (n=281), identifying obesity as one of the predictive independent risk factors for severe COVID-19 infection and respiratory disease in pediatric patients [14]. Common hospital complications included acute respiratory distress syndrome and AKI [14]. As recognized by Fernandes et al., these effects could be further studied on a minority population, which has shouldered a disproportionate burden during the COVID-19 pandemic [14].

Further studies with larger sample sizes may be able to indicate a stronger significance between underlying obesity and elevated inflammatory markers and MIS-C status. As pediatric obesity becomes increasingly prevalent, especially in South Texas [15], effective identification of risk factors for critical illness will improve COVID-19 hospital outcomes. Based on the findings of this single-center study, providers can better appreciate obesity as a significant risk factor in hospitalized COVID-19 pediatric patients. Obesity has been shown to be a significant independent risk factor for critical illness and hospital LOS [13], and our study also uncovers its role in MIS-C status, PICU admission, and longer PICU LOS.

Our single-center study had some limitations. The pediatric sample included patients admitted to the Children’s Hospital of San Antonio, and represented only that particular geographic region and respective demographic(s). The hospital and PICU admission criteria that we utilized may or may not be representative of the criteria employed by other hospital systems. Our pediatric sample was small (n=77) and efforts at a more focused analysis were limited by this small sample size. Statistical analysis was carefully carried out to reflect accepted models for analysis and feasible tests that could be satisfied by the appropriate sample size. Due to the retrospective nature of the study and a lack of access to clinic records, our study was limited in terms of determining obesity-related, pre-existing mild AKI with microalbuminuria in our cohort. Since the key objective of the study was to investigate the role of underlying obesity in COVID-19 presentations and outcomes at a tertiary care children’s hospital, the study did not analyze the association between outcomes of COVID-19 and adolescence and/or LGBTQ population and comorbidities other than obesity. In the future, we plan to assess these parameters, including the progression of the disease or any delay in accessing medical care among obese children.

## Conclusions

Pediatric obesity is becoming increasingly prevalent, and this single-center report captures the prevalence of childhood obesity in the context of the COVID-9 pandemic. Of the admitted COVID-19 patients, more than half had an obese BMI classification, and they were predominantly of Hispanic ethnicity. Obese BMI classification was found to be significant in terms of patient age distribution, comorbidities, MIS-C status, and PICU LOS. Hospital mortality in pediatric COVID-19 patients was low and consistent with other studies in the literature showing lower rates of mortality in children versus mortality in adult patients with COVID-19.

## References

[1] Sattar N, McInnes IB, McMurray JJ (2020). Obesity is a risk factor for severe COVID-19 infection: multiple potential mechanisms. Circulation.

[2] Sanchis-Gomar F, Lavie CJ, Mehra MR, Henry BM, Lippi G (2020). Obesity and outcomes in COVID-19: when an epidemic and pandemic collide. Mayo Clin Proc.

[3] Kompaniyets L, Goodman AB, Belay B (2021). Body mass index and risk for COVID-19-related hospitalization, intensive care unit admission, invasive mechanical ventilation, and death - United States, March-December 2020. MMWR Morb Mortal Wkly Rep.

[4] Macias Gil R, Marcelin JR, Zuniga-Blanco B, Marquez C, Mathew T, Piggott DA (2020). COVID-19 pandemic: disparate health impact on the Hispanic/Latinx population in the United States. J Infect Dis.

[5] Ludvigsson JF (2020). Systematic review of COVID-19 in children shows milder cases and a better prognosis than adults. Acta Paediatr.

[6] Mehta NS, Mytton OT, Mullins EW (2020). SARS-CoV-2 (COVID-19): what do we know about children? A systematic review. Clin Infect Dis.

[7] Alsohime F, Temsah MH, Al-Nemri AM, Somily AM, Al-Subaie S (2020). COVID-19 infection prevalence in pediatric population: etiology, clinical presentation, and outcome. J Infect Public Health.

[8] Stewart DJ, Hartley JC, Johnson M, Marks SD, du Pré P, Stojanovic J (2020). Renal dysfunction in hospitalised children with COVID-19. Lancet Child Adolesc Health.

[9] Kari JA, Shalaby MA, Albanna AS, Alahmadi TS, Alherbish A, Alhasan KA (2021). Acute kidney injury in children with COVID-19: a retrospective study. BMC Nephrol.

[10] Panigrahy N, Policarpio J, Ramanathan R (2020). Multisystem inflammatory syndrome in children and SARS-CoV-2: a scoping review. J Pediatr Rehabil Med.

[11] Antúnez-Montes OY, Escamilla MI, Figueroa-Uribe AF (2021). COVID-19 and multisystem inflammatory syndrome in Latin American children: a multinational study. Pediatr Infect Dis J.

[12] Bhalala US, Gist KM, Tripathi S (2022). Characterization and outcomes of hospitalized children with coronavirus disease 2019: a report from a multicenter, viral infection and respiratory illness universal study (coronavirus disease 2019) registry. Crit Care Med.

[13] Tripathi S, Christison AL, Levy E (2021). The impact of obesity on disease severity and outcomes among hospitalized children with COVID-19. Hosp Pediatr.

[14] Fernandes DM, Oliveira CR, Guerguis S (2021). Severe acute respiratory syndrome coronavirus 2 clinical syndromes and predictors of disease severity in hospitalized children and youth. J Pediatr.

[15] Foster BA, Maness TM, Aquino CA (2017). Trends and disparities in the prevalence of childhood obesity in south Texas between 2009 and 2015. J Obes.

[16] (2022). Centers for Disease Control and Prevention. A SAS program for the 2000 CDC growth charts (ages 0 to 20 years). the.

[17] (2022). Centers for Disease Control and Prevention. A SAS program for the WHO growth charts (ages 0 to <2 years). https://www.cdc.gov/nccdphp/dnpao/growthcharts/resources/.

[18] Ayalon I, Woo JG, Basu RK, Kaddourah A, Goldstein SL, Kaplan JM (2020). Weight as a risk factor for mortality in critically ill patients. Pediatrics.

[19] Gonçalves JP, Severo M, Rocha C, Jardim J, Mota T, Ribeiro A (2015). Performance of PRISM III and PELOD-2 scores in a pediatric intensive care unit. Eur J Pediatr.

[20] Soler YA, Nieves-Plaza M, Prieto M, García-De Jesús R, Suárez-Rivera M (2013). Pediatric Risk, Injury, Failure, Loss, End-Stage renal disease score identifies acute kidney injury and predicts mortality in critically ill children: a prospective study. Pediatr Crit Care Med.

[21] (2022). Centers for Disease Control and Prevention. Multisystem inflammatory syndrome (MIS-C). https://www.cdc.gov/mis-c/index.html.

[22] Daniel WW, Cross CL (2018). Biostatistics: A Foundation for Analysis in the Health Sciences.

[23] Agresti A (2018). An Introduction to Categorical Data Analysis. https://books.google.co.in/books/about/An_Introduction_to_Categorical_Data_Anal.html?id=vSnvzgEACAAJ&source=kp_book_description&redir_esc=y.

[24] RStudio Team (2021 (2022). RStudio Team. http://www.rstudio.com/.

